# NFKB1 rs28362491 and pre-miRNA-146a rs2910164 SNPs on E-Cadherin expression in case of idiopathic oligospermia: A case-control study

**Published:** 2018-04

**Authors:** Matem Tunçdemir, Güven Yenmiş, Kübra Tombultürk, Hülya Arkan, Tuğba Soydaş, Rasit Burak Tek, Özlem Altıntaş, Hamdi Özkara, Gönül Kanıgür-Sultuybek

**Affiliations:** 1 *Department of Medical Biology, Cerrahpasa Medical Faculty, Istanbul University, Istanbul, Turkey.*; 2 *Acıbadem Healthcare Services, Acıbadem LABGEN Genetic Diagnostic Center, Acibadem University Kerem Aydınlar Campus, Icerenkoy Kayisdagi Istanbul, Turkey.*; 3 *Medical Laboratory Techniques, Vocational School of Health Services, Istinye University, Istanbul, Turkey.*; 4 *Department of Urology, Cerrahpasa Medical Faculty, Istanbul University, Istanbul, Turkey.*; 5 *Department of Medical Biology and, Genetics, Medical Faculty, Istanbul Aydin University, Istanbul, Turkey.*

**Keywords:** E-Cadherin, Oligospermia, Male infertility, Nuclear factor kappa B, Mir-146a

## Abstract

**Background:**

A notable proportion of idiopathic male infertility cases is accompanied by oligozoospermia; and yet, the molecular mechanisms of fertilization problem underlying this defect are still unclear. Epithelial cadherin has been involved in several calcium-dependent cell-to-cell adhesion events; however, its participation in gamete interaction has also not been fully investigated.

**Objective:**

The aim was to investigate the changes in the expression of E-cadherin, based on the frequency of Single nucleotide polymorphisms in Nuclear Factor Kappa-B 1 and pre-mir-146a in oligospermic men.

**Materials and Methods:**

In this case-control study, semen and blood samples of 131 oligospermic men as the case group and 239 fertile healthy men as the control group were analyzed. Variants single nucleotide polymorphisms rs28362491 and rs2910164 were performed using polymerase chain reaction-restriction fragment length polymorphism method and E-cadherin expression were determined by immunoprecipitation studies.

**Results:**

ins/ins genotype of rs28362491 was determined as a risk factor for idiopathic oligospermia by 1.73 times (p=0.0218), whereas no significant differences were found between the groups concerning pre-mir-146a rs2910164 polymorphism (p=0.2274 in case of GC genotype and p=0.9052 in case of GG genotype). Combined genotype analysis results did not show any notable differences between the multiple comparisons of 28362491-rs2910164 in oligospermic men and control groups. In addition, E-cadherin expression of oligospermic men with ins/ins genotype was significantly lower than patients with del/ins genotype (p=0.0221). E-cadherin expression level was low in oligospermic men with respect to the control group in presence of ins/ins genotype of NFKB1 gene.

**Conclusion:**

These results suggest that ins allele prevents binding of surface proteins to spermatozoa, leading to a low affinity of sperm-oocyte interaction in oligospermic men.

## Introduction

Male infertility is a growing problem that affects couples worldwide. While nearly half of the infertility cases are attributed to male factors, 60% of them are idiopathic (1). Azoospermia or severe oligozoospermia is responsible for the major proportion of idiopathic male infertility cases. An interaction between sperm and egg is compulsory, but the molecular mechanisms of direct binding and fusion reactions of sperm-egg membrane proteins are poorly understood. Focusing on this missing part would be useful in understanding at least some of the factors associated with fertilization defects. One of the candidate genes in spermatogenesis whose failure may cause male infertility is Nuclear Factor Kappa-B (NF-KB), a master regulator of immunity and inflammation, anti-apoptosis, and cell proliferation responses (2, 3). The variants on the promoter sites of NF-KB coding gene, NFKB1, have been studied in connection with the numerous diseases (4). A functional polymorphism, -94 ATTG insertion/deletion (ins/del) (rs28362491), located in between two putative key promoter regulatory elements of NFKB1 destroys a transcription factor binding site; hence leading to differential expression (5). Previously, Bianco and his colleagues demonstrated a positive association of this polymorphism with severe endometriosis and idiopathic infertility (6). 

As a proof of their cooperation, the stimulation of NF-KB transcriptional activity is triggered by an E-cadherin siRNA (7). E-cadherin interacts homophilically with E-cadherin molecules of adjacent cells, and alfa catenin proteins are proven to be good indices of infertility (8). Hernandez Gifford and his colleagues suggested anti-cadherin/ anti-catenin antibodies for male contraception (9) and, Purohit and co-workers reported E-cadherin presence in both human spermatozoa, and also its absence on the head of oligospermic individuals; however, the precise mechanisms of recognition and fusion remain unclear (10). 

Numerous miRNA expressions have been verified in testis; suggesting that miRNAs may play substantial roles in spermatogenesis (11). MiR-146a is transcriptionally induced by NF-KB in response to activation of innate immune signaling (12). More recently, the pre-mir-146a has also received considerable attention; single nucleotide polymorphisms (SNP) of pre-mir-146a leads to a less mature miRNA expression which in turn affects the transcription of target genes such as NFKB1 (13).

Based on all these findings, several genes such as NFKB1, pre-mir-146a, and E-cadherin, can be considered as prominent factors in spermatogenesis and fertilization. We hypothesized that NFKB1 variants and its regulator pre-mir-146a and the possible failures of sperm membrane-associated proteins such as E-cadherin could be potentially involved in the pathogenesis of infertility of oligospermia. 

Therefore, the genotype frequencies of SNPs, rs28362491 (-94 ATTG ins/del, NFKB1) and its regulator miRNA, pre-mir-146a were compared in a single and combined genotype analysis, and their association with the expression of E-cadherin in oligospermic patients and fertile controls were determined.

## Materials and methods

**Study population**

The study population consisted of men who attended the Andrology Clinics of Urology Department, Cerrahpasa Medical Faculty of Istanbul University for infertility evaluation between April 2012 and February 2014.

One hundred thirty-one oligospermic men who were unsuccessful to achieve pregnancy with their partners after intercourse without contraception for 1 yr, were evaluated as infertile-case group. They underwent an andrological examination and at least two semen samples were analyzed for each case. Semen analysis was performed according to World Health Organization Laboratory Manual (14). Sperm morphology was assessed using a strict criterion (15). 

All men with the history of epididymitis, orchitis, cryptorchidism, varicocelectomy, as well as men under treatment with spermatogenesis-impairing medication and having Y chromosome microdeletion or infertile partner (infertile female factor) were excluded from the study. The control group including 239 men where chosen among the patients who attended to the clinic for other urological diseases and they had fathered a child in a 2 yr period and also all had a normal andrological examination. Men were requested to abstain from sexual activity for 3-4 days before the semen collection.The semen materials were collected in a private room of the andrology clinic within the laboratory. The samples were obtained by masturbation and ejaculated into a sterile container. In case of less than 10×10^6^ mL sperm concentration, serum follicle stimulating hormone and total testosterone levels were measured. 

**Blood samples and DNA isolation**

Peripheral blood samples (2 mL) were collected in K3 tubes containing EDTA. After collection, genomic DNA is extracted using Roche DNA purification kit (Roche Diagnostics GmbH, Mannheim, Germany; Product No.11796828001) according to the manufacturer's instructions. Concentration and purity of DNA were measured by a NanoDrop™ spectrophotometer and determined by 260/280 nm OR. DNA samples with 260/280 OR of 1.8±0.1 were included in the study.

**Genotyping**

SNPs NFKB1-94 ins/del ATTG rs28362491 and pre-mir-146a rs2910164 were determined both in the case and control groups by polymerase chain reaction (PCR)-restriction fragment length polymorphism method. Applied Biosystems XP Cycler thermal cycler was used for PCR method. PCR conditions for rs28362491 were set at 95^o^C for 2 min initial denaturation, followed by 35 cycles of 95^o^C for 30 denaturations, 60^o^C for 30 sec annealing primers, 72^o^C for 1 min extension and finally 72^o^C 5 min. On the other hand, PCR conditions for rs2910164 were 5 min at 94^o^C followed by 35 cycles of 1 min at 94^o^C, 1 min at 58^o^C and 2 min at 72^o^C, with a final step at 72^o^C for 20 min to allow a whole extension of all PCR fragments. PCR primers were, forward primer (F), -5'-TGG GCA CAA GTC GTT TAT GA-3', reverse primer(R)- 5'-CTG GAG CCG GTA GGG AAG-3' for rs28362491; and (F) 5'- ATG GGT TGT GTC AGT GTC AGA GTC-3', (R) 5'-TGC CTT CTG TCT CCA GTC TTC CCA-3' for rs2910164. 

**Figure 1:**
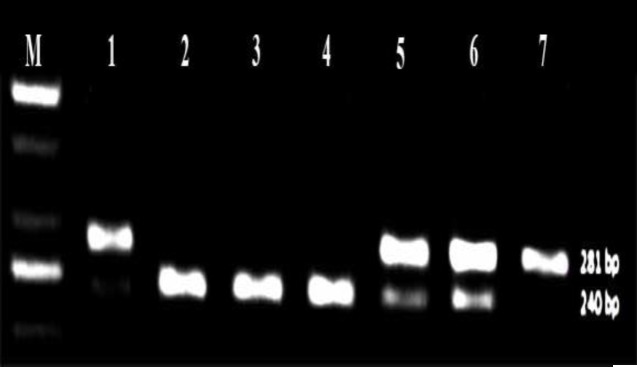
The enzyme digestion pattern of rs28362491. M is 50-bp size marker; Lane 5 and 6 are heterozygous ins/del ATTG; Lane 2, 3 and 4 are homozygous ins/ins ATTG; Lane 1 and 7 are del/del ATTG homozygous

**Figure 2:**
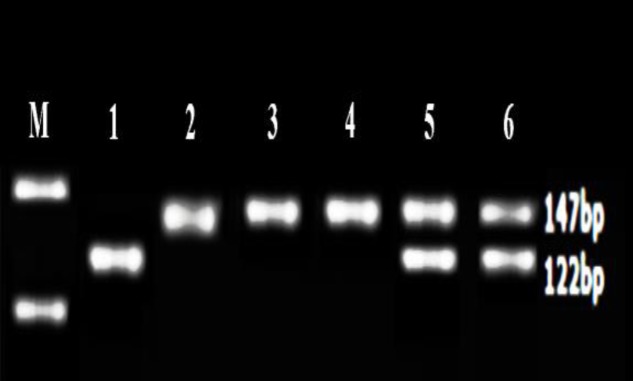
The enzyme digestion pattern of rs2910164. M is 50-bp size marker; Lane 1 is wild-type homozygous alleles (C/C); Lane 2, 3 and 4 are mutant type homozygous alleles (G/G); Lane 5 and 6 are heterozygous alleles (C/G

Thermo Scientific Easycast B1A device and Syngene gel imaging and analysis system were used for restriction fragment length polymorphism method. For detection of rs28362491 polymorphism, PCR product was digested with 3 unit of restriction enzyme PfIMI (10 U/µl, Fermentas). Sac I (20,000 U/ml, NEB) was used for rs2910164 polymorphism. In case of PfIMI restriction,the PCR product of 281 bp remained undigested.( the wild-type (deletion) genotype). The insertion genotype were seen as two fragments of 240 bp and 45 bp. In heterozygotes all three bands were observed (Figure 1). In case of Sac I restriction for rs2910164 polymorphisma single 147 bp fragment for the mutated type homozygous alleles (GG genotype); two fragments of 122 and 25 bp for the wild-type homozygous alleles (CC genotype); and three fragments of 147, 122, and 25 bp for the heterozygous alleles (GC genotype) were observed(Figure 2).

**Immunodetection of E-Cadherin by Immunocytochemistry**

Briefly, sperms were washed and resuspended in fixed 10% neutral buffered formalin for 20 min at room temperature. Histostain-Plus Bulk Kit (Invitrogen® 2nd Generation LAB-SA Detection System, Camarillo, CA) and rabbit polyclonal E-cadherin (Santa Cruz Biotech., sc-7870, 1:50 dilution) antibodies were used with the Streptavidin-Biotin-Peroxidase method in order to stain the smears onto a clean glass slide by the immunocytochemistry stain procedure offered in the kit. The sections were incubated with a substrate-chromogen solution (AEC; Invitrogen, San Francisco CA, USA) for 5-6 min. Following the counterstaining with methyl green, sections were incubated following the above procedure to determine the specificity of immunostaining, except for the omission of incubation with the primary antibody. The control serum was also used as a control instead of primary antibody.

**Evaluation of the immunocytochemical stain signals**

**Figure 3:**
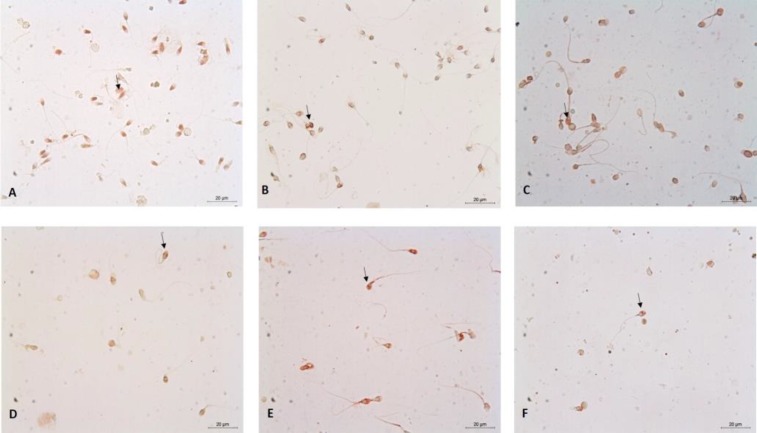
Immunoreactivity of E-cadherin. Control groups ins/ins (A), del/ins (B) and del/del (C). Case group; ins/ins (D), del/ins (E) and del/del (F). Red staining cells: E-cadherin (+) sperms (↑). Background: Methyl green- Bar: 20µm

E-cadherin immunoperoxidase staining; under a microscope at 40× magnification, 15 areas were selected randomly and immunostained semen samples were evaluated semiquantitatively and scored from 1+ to 3+ (where 1+refers to weak, and 3+refers to strong immunopositivity) (Figure 3), as described previously (16).

**Ethical consideration**

The Institutional Ethics Committee of Istanbul University Cerrahpasa Medical School approved the study protocol in accordance with the Helsinki declaration and written informed consent of all participants (case and control group) were gathered. (Ethics Committee Approval Number 83045809/14878). Participants were informed that the knowledge collected would be kept anonymous and participation was totally voluntary.

**Statistical analysis**

Statistical analysis was performed using the SPSS software (Statistical Package for the Social Sciences, version 18.0, SPSS Inc, Chicago, Illinois, USA) and p<0.05 (two-sided) was used as the criterion of statistical significance. Hardy-Weinberg equilibrium was determined using chi-square (χ^2^) analysis. Genotype and allele frequencies were compared between cases and controls by Yates’s Continuity Correction, Fisher’s Exact Test and χ^2^ analysis. The effects of any difference between allelic and genotype distribution were considered by odds ratio (OR) and respective 95% confidence intervals (CIs). Mann-Whitney U test for the analysis of clinical features, and One-way ANOVA and Tukey's HSD Post hoc tests for the analysis of E-cadherin were performed.

## Results

The study included 239 controls (30.75±9.36 yr) and 131 cases (33.03±5.24 yr). There were no significant differences in terms of age, however, differences in sperm concentration, sperm motility, and sperm morphology were present, as expected. In addition, testicular volume right and left values had no differences (Table I).

The distribution of the genotype and allele frequencies of SNPs (rs28362491, rs2910164) studied is shown in Table II. All alleles and genotype frequencies were in the range of Hardy-Weinberg equilibrium. The frequencies of genotypes of NFKB1-94ins/delATTG in case group were found to be significantly different with respect to control group. The statistical analysis of the data using χ^2^-test showed that the ins/ins genotype of -94ins/del ATTG polymorphism was associated with a significantly increased risk of idiopathic oligospermia when compared to the ins/del genotype (OR=1.731, 95% CI: 1.08-2.77, p=0.021). However, the ins allele frequency in idiopathic oligospermic cases was not significantly different from that of the control group (p=0.179). 

We also tested the association of pre-mir-146a rs2910164 with susceptibility to male infertility with idiopathic oligospermia. As shown in Table II, there are no statistically significant differences in the allele frequency and genotype distribution of the pre-mir-146a rs2910164 polymorphisms (p=0.2274 in case of GC genotype and p=0.9052 in case of CC genotype) between oligospermic men and control groups. In addition, we examined the combined genotype analysis of NFKB1 and pre-mir-146a polymorphisms. There were no significant differences between multiple comparisons of rs28362491 and rs2910164 in oligospermic men and control group (See Table III).

According to E-cadherin expression of both oligospermic patients and control groups, E-cadherin expression in oligospermic patients (1.66±1.02) was significantly lower than the control group (2.85±1.14) (p<0.01) (Figure 3). Furthermore, when E-cadherin expression was compared to the genotypes of NFKB1 rs28362491 polymorphism in oligospermic men and controls, there was a significant difference among the genotypes. According to Figure 3, we observed that the patients with del/ins genotype had higher E-cadherin expression than the patients with ins/ins and del/del genotype (p=0.0221) (Table IV). In other words, del/ins rs28362491 genotype could have a protective role in the case of fertilization in idiopathic oligospermia, whereas ins/ins and del/del genotype of NFKB1 could be a risk factor.

**Table I:** Clinical feature of (cases) and controls included in the study

		**Case group (n=131)**	**Control group (n=239)**	**p-value**
Age (yr)		33.03 ± 5.24	30.75 ± 9.356	0.0783
Sperm concentration (×10^6^/ml)		4.79 ± 4.12	34.93 ± 26.40	<0.0001
Sperm motility (%)		24.62 ± 20.56	41.89 ± 19.38	0.0016
Sperm morphology (%)		3.56 ± 3.38	7.556 ± 4.878	0.0021
Testicular volume				
	Right (ml)	13.63 ± 6.05	15.61 ± 9.562	0.8693
	Left (ml)	14.04 ± 6.27	15.72 ± 8.870	0.9011

Data are shown as mean±SD

Groups were compared by Mann Whitney U test

**Table II:** Distribution of genotype and allele frequencies of SNPs rs28362491 and rs29101164 in (case group) and controls

**rs29101164 **	**Case group (n=131)**	**Control group (n-239)**	**OR (%95 Cl)**	**p-value**
GG	73 (55.7)	120 (50.2)	1	Ref
GC	50 (38.2)	108 (45.2)	1.314 (0.8429-2.048)	0.2274
CC	8 (6.1)	11 (4.6)	0.8365 (0.3215-2.176)	0.9052
Dominant model ((GG+ GC) vs CC)			0.7418 (0.2906-1.893)	0.7034
Recessive model (GG vs (GC + CC))			1.248 (0.8135-1.915)	0.3097
G allele	196 (74.8)	348 (72.8)	1	Ref
C allele	66 (25.2)	130 (27.2)	1.109 (0.7863-1.565)	0.5543
rs28362491				
ins/ins	51 (38.9)	67 (28)	1	Ref
del/ins	61 (47.3)	141 (59)	1.1731 (1.081-2.773)	0.0218
del/del	18 (13.8)	31 (13)	1.311 (0.6604-2.603)	0.5469
Dominant model ((ins/ins+del/ins) vs del/del)			0.9356 (0.5011-1.747)	0.9613
Recessive model(ins/ins vs (del/ins+del/del))			1.637 (1.043-2.568)	0.0315
ins allele	164 (62.6)	275 (57.5)	1	Ref
del allele	98 (37.4)	203 (42.5)	1.235 (0.9068-1.683)	0.1799

Data presented as n (%). Groups were compared by χ2 analysis and Yates’s Continuity Correction.

The differences resulting from the genotypes were determined by proceeding χ2 analysis and Yates’s Continuity Correction.

**Table III:** Combined genotypes of rs8362491 and rs29101164 in case group and fertile controls

**NFKB1- mir146a combined genotypes**	**Case group (n=131)**	**Control group (n-239)**	**OR (%95 Cl)**	**p-value**
ins/ins/GG	19 (16.4)	27 (11.3)	1	Ref
del/ins/GG	34 (29.3)	76 (31.8)	1.573 (0.7711-3.209)	0.2113
del/del/GG	11 (9.5)	17 (7.1)	1.088 (0.4167-2.838)	0.9421
ins/ins/GC	12 (10.3)	37(15.5)	2.170 (0.9029-5.214)	0.1265
del/ins/GC	24 (20.7)	59 (24.7)	1.730 (0.8132-3.680)	0.2169
del/del/GC	9 (7.7)	12 (5)	0.9383 (0.3300-2.668)	0.8828
ins/ins/CC	3 (2.6)	5 (2.0)	0.7037 (0.1279-3.871)	0.9730
del/ins/CC	3 (2.6)	6 (2.5)	1.407 (0.3123-6.342)	0.9407

Data presented as n (%).

del/del/CC combined genotype was not added to statistical analysis due to inadequate number of individuals. The differences resulting from the genotypes were determined by proceeding χ2 analysis, fisher’s exact test and Yates’s continuity correction.

**Table IV.** Evaluation of E-cadherin expression according to the genotypes of NFKB1 rs28362491 in men Case group

**Genotypes of NFKB1 -94 ins/del ATTG**	**Case group, n (%)**	**E-cadherin expression**
ins/ins	51 (39)	1.28 ± 0.90
del/ins	62 (47)	2.5 ± 1.08 *
del/del	18 (14)	1.21 ± 0.56

Groups were compared by One-way ANOVA and Tukey's HSD Post hoc tests.

Data are shown as Mean ± SD.

* p= 0.0221 ins/ins vs. del/del.

**Table V:** World Health Organization. WHO laboratory manual for the examination and processing of human semen (2010) (17)

**Criteria minimum semen parameters**	**WHO 2010**
Semen Volume (mL)	1.5 (1.4-1.7)
Sperm Concentration (10^6^)	15 (12-16)
Sperm Motility (% Total)	40 (38-42)
Sperm Morphology (% normal forms)	4 (3.0-4.0)
Sperm Viability (% viable)	58 (55-63)

## Discussion

Male infertility is a worldwide problem in which testis-specific gene anomalies, impaired spermatogenesis, and reduction of fertilization may be involved. Fertilization is a fascinating event in sexual reproduction which requires the fusion of haploid sperm and egg so creating a genetically distinct, new diploid organism. Appropriate cell contacts (sperm and egg in this case) are necessary for communication between cells, movements and also for segregation of groups of cells to form tissues and organs. There have been many studies with several experimental models to identify the nature of the molecules involved in gamete recognition, yet it still remains unclear. An understanding of the roles of cell adhesion molecules is, therefore, important in analyzing fertilization process.

Cadherins, calcium-dependent cell surface proteins that mediate cell adhesion mainly by homophilic binding, are believed to be substantially important for cell-cell interactions and considered as one of the candidate proteins for fertilization process (17). Ponce and Caballero reported the involvement of E-cadherin in cell-to-cell adhesion events during fertilization (18). E-cadherin, but not N-cadherin, is present in spermatozoa lysates of fertile subjects whereas it is absent in oligospermic subjects; so it probably plays a role in the recognition process preceding gamete fusion (19). In the present study, we found that E-cadherin expression in oligospermic patients was significantly lower than the control group. According to our previous publication, the existence of insertion allele of NFKB1 rs28362491 is correlated with low E-cadherin and fibronectin levels in the normospermic patients (20).

NF-KB, as a transcription factor, is responsible for the regulation of many other genes in case of disease progression; therefore, variants in the genes coding for the NF-KB protein may potentially be involved in disease development. According to Delfino & Walker and Pentikainen; during all stages of spermatogenesis (I-XIV), subunits of these proteins p50 and p65 were present and active in the nucleus of Sertoli cells. In addition, NF-kB has a role in the regulation of stage-specific gene expression of male infertility (21, 22). Although the mechanism behind NFKB1 in relation to disease susceptibility remains unclear, the presence of a 4 bp deletion (-94delATTG-rs28362491) in NFKB1 promoter region resulted in the loss of binding to nuclear proteins, eventually leading to reduced promoter activity (5). 

SNP rs28362491 in promoter sites of NFKB1 has been reported to have a positive association with both moderate and severe endometriosis caused infertility, and idiopathic female infertility (7). Ranganathan and coworkers showed that decrease of NFKB1 expression leads to poor sperm production (23). However, in the literature, there are no studies that illuminate the relationship between oligospermic male infertility and promoter site variations of NFKB1 rs28362491. For this purpose, we hypothesized that SNPs in promoter sites of rs28362491 in the NFKB1 gene might be involved in the pathogenesis of oligospermic male infertility in a Turkish population. We determined that the risk of disease for oligospermic infertile patients with ins/ins genotype is increased by 1.17 times.

It has recently been reported that miRNAs’ dysregulation plays prominent roles in the development of many diseases namely male reproductive disorders.

However, the role of miRNAs in male infertility and miRNA -mediated gene regulation in spermatogenesis have not been clearly elucidated. Lian and his colleagues did the first study which reported an alteration of miRNA expression in the testis of patients with non-obstructive azoospermia (24). In fact, numerous miRNAs are primarily expressed in the mouse testis, suggesting their inevitable role in spermatogenesis (25). Wang and co-workers reported that the level of 7 miRNAs (miR-509 -5p, miR-34c-5p, miR-146b-5p, miR-122, miR-181a, miR-374b, and miR-513a-5p) was higher in asthenozoospermia and lower in azoospermia and, compared to control. It is also noted that pre-mir-146a, which is an NF-kB target gene, may play an essential role in the development of infertility (26). 

Many studies reported the essential role of pre-mir-146a, an NF-kB target gene, in elavating the inflammatory response in inflammatory diseases (12). Pre-mir-146a SNPs can modify the expression level of mature miRNA-146a hence have functional importance .The SNP rs2910164 (G/C), for example, leads to a decrease in the total amount of mature miRNA, via affecting the transcription of target genes and the pathogenesis of inflammatory diseases such as rheumatoid arthritis (27). Therefore, we investigated the single or combined effects of NFKB1 rs28362491 and tested if its regulator miRNA, pre-mir-146a rs2910164 is related to the susceptibility to oligospermia in a Turkish population. According to Table 2 and Table 3, no significant differences were found in the single pre-mir-146a rs2910164 or the combined effects of NFKB1 rs28362491–pre-mir-146a rs2910164.

In the light of these data; variations in NFKB1 gene, which has an impact on gene expressions and adhesion molecules including E-cadherin, would be affinitive candidate factors of male infertility risk. Hereby, we hypothesized that the relationship between E-cadherin expression and oligospermia risk could be changed by rs28362491, that is, a common functional polymorphism in NFKB1 gene. In this study, the expression of E-cadherin was determined and compared to the genotypes of SNP rs28362491 in oligospermia as its pathophysiology is still unknown. We observed that oligospermic patients with ins/ins genotype had significantly lower E-cadherin expression when compared to control group (p<0.05). 

Hence, E-cadherin on the surface of sperm cells of oligospermic patients was decreased by the involvement of ins/ins genotype compared to del/ins one. In other words, the existence of ins/ins genotype may decrease the affinity of sperm-egg interactions and finally lead to infertility. Taken together, del/ins genotype may result in a higher adhesion molecule level and prevent infertility in contrast to ins/ins and del/del genotype. These results suggest that the wild homozygosity of SNP rs28362491 provides a risk factor for oligospermic male infertility in a Turkish population. Increased knowledge about the associations of SNPs and the biology of sperm membrane proteins improves our capacity to evaluate the diseases based on molecular experiments and to analyze the results in a significant manner. In this report, we investigated the relationship between gene variants, such as rs28362491, and E-cadherin expression which we thought were important in sperm-egg interactions during fertilization to bring a new insight to the oligospermic male infertility problem in a Turkish population. However, these findings should be confirmed in an independent sample and in different populations.

## Conclusion

We found that existence of ins allele of rs28362491 was correlated with increased risk for development of male infertility. Moreover, ins/ins and del/del genotypes in the promoter region, NFKB1 is likely to play a role in the susceptibility to oligospermic male infertility in the context of low E-cadherin expression.
